# Subfertility in mice harboring a mutation in βB2-crystallin

**Published:** 2007-03-14

**Authors:** Kevin M. DuPrey, Kimberly M. Robinson, Yan Wang, Jennifer R. Taube, Melinda K. Duncan

**Affiliations:** Department of Biological Sciences, University of Delaware, Newark, DE

## Abstract

**Purpose:**

βB2-crystallin is one of the most abundant proteins of the adult ocular lens of mammals although it is expressed at lower levels in several extralenticular locations. While mutations in βB2-crystallin are known to result in lens opacities, alterations in tissues besides the lens have not been previously investigated in these mutants. Since we found mice harboring the *Crybb2^Phil^* mutation bred poorly, here we assess the contribution of βB2-crystallin to mouse fertility and determine the expression pattern of βB2-crystallin in the testis.

**Methods:**

The expression pattern of βB2-crystallin in the testis was analyzed by rt-PCR, western blotting, and immunohistochemistry. The fecundity of wildtype and *Crybb2^Phil^* mice was analyzed by quantitative fertility testing. The morphology of testes and ovaries was assessed by hematoxylin and eosin staining.

**Results:**

In the mouse testis, βB2-crystallin mRNA is found at low levels at birth, but its expression upregulates in this tissue as the testis is primed to initiate spermatogenesis. Western blotting detected βB2-crystallin protein in sperm obtained from mice, cattle, and humans while immunolocalization detected this protein in developing sperm from the spermatocyte stage onward. Male and female mice homozygous for a 12 nucleotide inframe deletion mutation in βB2-crystallin are subfertile when analyzed on a Swiss Webster derived background due to defects in egg and sperm production. However, mice harboring the same mutation on the C57Bl/6 genetic background did not exhibit any defects in reproductive function.

**Conclusions:**

βB2-crystallin is expressed in developing and mature sperm and mice of both sexes harboring the Philly mutation in the βB2-crystallin gene are subfertile when analyzed on a Swiss Webster genetic background. While these data are suggestive of a role for βB2-crystallin in fertility, definitive determination of this will await the creation of a βB2-crystallin null mouse.

## Introduction

The ocular lens is a transparent tissue that accumulates cytoplasmic proteins at concentrations approaching 450 mg/ml in order to reach the refractive index necessary to focus light onto the retina [1,2]. These abundant proteins are named as crystallins if their individual concentration approaches 5% or more of the water soluble proteins of the lens of any species [3]. In many cases, crystallins are either transcribed from the same gene as a common metabolic enzyme or have arisen from a recent gene duplication from such a gene (the taxon specific crystallins) [4]. Further, all vertebrates have two genes for α-crystallins which are members of the small heat shock protein family. One of these, αB-crystallin, is expressed at appreciable levels in multiple tissues besides the lens where it has been shown to be protective against and/or induced by numerous stress responses [5].

All vertebrates also express members of the β/γ-crystallin superfamily at high levels in their lenses [6]. These proteins are defined by sequence similarities that result in the presence of two or more "β/γ-crystallin Greek key motifs", a characteristic anti-parallel β-sheet conformation, in their tertiary structure [7]. Mutations in β- or γ-crystallin genes cause cataracts in both mice and humans [8] showing that they are required to maintain lens transparency. However, even though many β/γ-crystallins are expressed at sites outside of the lens [9,10], and a prokaryotic member of this family, Protein S, is a calcium binding protein that forms the spore coat of the colonial prokaryote *Myxococcus xanthus* [11], the extralenticular functions of vertebrate β/γ-crystallins are not known.

βB2-crystallin is a major component of the postnatal vertebrate lens [12-14] and it is the most abundant β-crystallin of adult lenses in many species. βB2-crystallin is also the most thermally stable [9,15] and resistant to modification [16] of all of the β-crystallins and has been hypothesized to be crucial to maintain the solubility of other crystallins in the adult lens. It has been previously reported that βB2-crystallin is expressed in many extra-lenticular sites including the retina, brain, and testis [9]. Recently, βB2-crystallin was shown to be localized in retinal ganglion cells (RGCs) in the retina [17] and the protein was reported to be secreted, then internalized by RGCs to promotes neurite outgrowth during retinal regeneration [18].

Mice exhibiting autosomal dominant cataracts caused by a 12 nucleotide in frame deletion removing four amino acids from the fourth Greek key of βB2-crystallin (*crybb2^phil^*) arose spontaneously in an outbred Swiss Webster breeding colony in the late 1970s [19,20] and were maintained as homozygotes for many years in a small breeding colony until being cryopreserved. Once recovered, *crybb2^phil^* mice were found to be poor breeders and here we show that both males and females are subfertile as compared to Swiss Webster outbreds due to defective sperm and egg production. However, subfertility was not observed in male *crybb2^phil^* mice backcrossed onto a C57Bl/6NHsd genetic background suggesting that the poor reproductive performance of the original mutants is either strain dependent or due to inbreeding depression of the founding outbred Swiss Webster stock.

## Methods

### Mice

All experiments using animals were approved by the University of Delaware Institutional Animal Care and Use Committee and conform to the National Research Council Guide for the Care and Use of Laboratory Animals. Swiss-Webster and C57Bl/6NHsd breeding pairs as well as Hsd:ICR (CD-1) females were acquired from Harlan-Sprague Dawley (Indianapolis, Indiana). Homozygous STOCK *Crybb2^Phil^* mice on a Swiss Webster derived background were obtained from the National Institute of Health, Animal Genetics Resource, Veterinary Resources Program (Bethesda, MD). Swiss-Webster and homozygous STOCK *Crybb2^Phil^* mice were cross-bred to generate heterozygous *Crybb2^Phil^* F1 progeny. The *Crybb2^Phil^* mutation was moved onto the C57Bl/6NHsd genetic background by 8-10 generations of backcrossing followed by intercrossing to reestablish the lines as homozygous for *Crybb2^Phil^*. The presence of the *Crybb2^Phil^* allele was assessed by preparing DNA from tail biopsies using the PureGene Tissue and Mouse Tail kit (Gentra Systems, Minneapolis, MN) followed by PCR analysis using primers 5'- CTA CCG TGG GCT GCA GTA CCT GC -3' and 5'- GTG GAA GGC ACC TCG CTG GTG C -3' under the cycling conditions (95 °C, 30 s initial denaturation, followed by 30 cycles of 94 °C, 30 s; 60 °C, 30 s; 72 °C, 30; s, a 10 min extension at 72 °C, then a 4 °C hold). This yields a product of 132 nucleotides from the *Crybb2^+^* allele and one of 120 nucleotides from the *Crybb2^Phil^* allele which resolve well by electrophoresis on 10% polyacrylamide gels.

### Animal husbandry

All mice were maintained and bred in the University of Delaware animal facility under a 14/10 h light/dark cycle. Quarterly serological monitoring is conducted in every animal room on sentinel animals for Sendai, mouse hepatitis, pneumonia, minute, polyoma, epizootic diarrhea, pneumonitis, mouse parvo, ectromelia, reovirus, and lymphocytic choriomeningitis viruses as well as *Mycoplasma* species and *Helicobacter* using the Charles River Laboratories Health Monitoring Service (Wilmington, MA). The colony has tested negative for all of these pathogens for over the past six years. Monitoring for internal and external parasites is also performed and environmental monitoring of walls and floors for bacterial contamination is completed monthly. Mice were housed in open top cages (Ancare; Belmore, NY) with Beta-chip bedding (Harwood Laboratory Bedding Northeastern Products; Warrenburg, NY) changed at least once per week. Breeding mice were fed a high-energy, 19% fat variety of LabDiet, 5015 Mouse Diet (PMI Nutrition International, Richmond, IN) while nonbreeding mice were fed LabDiet, 5P00 Prolab RMH 3000. All mice are provided filtered drinking water ad libitum.

### Fertility testing

Fertility was assessed by setting up natural matings between a single male and female and recording the number of litters and pups born to each mating over a three month period. Fecundity was calculated as the total number of pups produced per mating per 30 day period. The data was analyzed statistically by unpaired Student's t-test, and subfertility was considered to be significant when p is <0.05.

### Assessment of testis and ovary

Testes and ovaries from age-matched mice were dissected and their gross anatomy observed under a Zeiss Stemi SV II Apo dissecting microscope fitted with a Nikon 4500 camera. Tissue weights were determined and the tissues fixed in Bouin's overnight, then transferred to 70% ethanol until embedment in paraffin. Six μm paraffin sections were created, then stained with hematoxylin and eosin using standard methods. Tissue morphology was observed under a light microscope, photographed, and transferred to Adobe Photoshop for comparative morphometric analysis. Statistical differences were determined by an unpaired Student's t-test.

### Sperm counts and motility assays

Cauda epididymides were minced in 2 ml of modified BWW medium (Irvine Scientific, Santa Ana, CA) with 3% BSA and sperm were allowed to swim out for ten min. For sperm counts, 50 μl of the sperm suspension was mixed with an equal volume of 2% paraformaldehyde and sperm counts assessed with a hemacytometer. For motility assays, 10 μl of the sperm suspension (or a 1:10 dilution of the suspension) was loaded on to the hemacytometer and the motility of 200 sperm assessed per sample. The data was analyzed statistically by an unpaired Student's t-test, and reduced sperm counts and motility were considered to be significant when p is <0.05.

### Apoptosis detection

Apoptotic activity in the testis was compared between STOCK *Crybb2^Phil^* and Swiss-Webster mice using an Apoptag Peroxidase Detection Kit S7100 with minor adjustments in staining time. Mouse testes were fixed in 4% paraformaldehyde for one h, transferred to 70% ethanol, embedded in paraffin, and sectioned. The slides were deparaffinized and digested with proteinase K, labeled with TdT-enzyme and anti-digoxigenin-horseradish peroxidase conjugate, stained with diaminobenzidine for 10 min, and counterstained with methyl green for 7 min. After dehydration through 100% N-butanol and citrus-based cleaning solvent, a drop of Cytoseal 60 (Electron Microscopy Sciences; Fort Washington, PA) was added and the slides coverslipped. Slides were observed under a light microscope and the number of apoptotic nuclei per seminiferous tubule recorded.

### RNA analysis

Total RNA was prepared from testes of Swiss-Webster mice at various developmental stages and adult lens using Promega's SV Total RNA Isolation System, according to the manufacturer's instructions. The forward primer (5'-TCT GAG GCC CAT CAA AGT GGA CAG CC 3') was designed over the splice site between exon 3 and exon 4 of the βB2-crystallin sequence and the reverse primer (5'-ACG CAC GGA AGA CAC CTT TTC CTG GTA 3') is the reverse complement of position 430-455 of the βB2-crystallin sequence, yielding a product size of 147 bp. RT-PCR reactions were performed using the One-Step RT-PCR System (Qiagen) with denaturation at 94 °C for 15 min, followed by 35 cycles of 94 °C for 30 s, 66 °C for 30 s, and 72 °C for 1 min, and final extension at 72 °C for 10 min. The RT-PCR products were electrophoretically separated on a 5% acrylamide gel and stained with ethidium bromide.

### Preparation of tissue extracts for SDS-PAGE and western blot analysis

Tissues were excised from euthanized adult mice and homogenized in 300 μl of RIPA buffer (285 μl of 1X PBS, 1% Igepal CA-630, 0.5% sodium deoxycholate, 0.1% SDS with 3 μl of 100 mu g/ml PMSF, 9 μl of 45 μg/ml aprotinin, and 3 μl of 1 mM sodium orthovanadate). The homogenates were cleared by centrifugation at 10,000x G for 10 min at 4 °C. The supernatant was then removed, the protein concentration determined by the DC Protein Assay (Bio-Rad, Hercules CA) and stored at -20 °C prior to analysis.

### Preparation of sperm for SDS-PAGE

Protein extracts from sperm were prepared as previously described [21]. Briefly, minced epididymides from age matched, sexual mature male Swiss-Webster and homozygous Crybb2^Phil^ mice, were incubated separately with gentle agitation at room temperature in 2 ml of sperm suspension buffer (50 mM Tris, 20 mM EDTA, 1 mM p-hydroxy-mercurobenzenzoate, 5 mM N-ethylmaleimide, 1 mM benzamidine, pH 7.2) for 10 min to disperse the sperm. The mouse sperm suspension was then centrifuged at 1,000 rpm for one min to pellet the tissues, and mouse sperm was collected by centrifuging at 2,500-3,000 rpm for ten min. Human sperm was provided from retired donors as a gift from the Andrology/Embryology Laboratory of the Oregon Health and Science University (Portland, OR). Bovine sperm was purchased from ABS Global (Deforest, WI).

### SDS-PAGE and western blot

Extracted protein from lens, sperm, and ovary from the Swiss-Webster mouse was analyzed using SDS-PAGE and western blot. Five nanograms of lens protein and 4x10^6^ sperm were diluted 1:1 with reducing sample buffer (0.125 M Tris-HCl, 0.1% SDS, 4% SDS, 20% glycerol, 32.4 mM DTT, and 1 μl of β-mercaptoethanol, pH 6.8) and were electrophoresed on a 12% SDS-polyacrylamide gel at 120 V. Proteins from the gel were transferred to a nitrocellulose membrane and blocked (1X TBS with Tween-20 [TBS/T] and 5% weight/volume nonfat dry milk) for one h at room temperature. Next, the membrane was probed with primary antibody (a generous gift from Dr. J. Fielding Hejtmancik, polyclonal rabbit anti-mouse, 1:2,000) in TBS/T-milk solution at 4 °C. The incubated membrane was washed three times for ten min each with 15 ml of TBS/T and then probed with secondary rabbit anti-mouse antibody (1:2,000 dilution, horseradish peroxidase [HRP] enzyme conjugated; Cell Signaling Technology, Beverly, MA) in TBS/T-milk solution. The membrane was again washed three times for ten min each with 15 ml of TBS/T and then immersed in chemiluminescence substrate solution (Cell Signaling Technology) for one min and exposed to x-ray film.

### Superovulation

Fifteen week old Swiss-Webster and homozygous STOCK *Crybb2^Phil^* females were superovulated by intraperitoneal injection of pregnant mare's serum gonadotropin followed 48 h later by five international units of human chorionic gonadotropin (hGC). The mice were sacrificed 14-18 h after the second injection, eggs isolated from the ampulla of the oviduct, and counted.

### Immunohistochemical studies

Testes were fixed in 4% parafomaldehyde for one h, rinsed in 1X phosphate buffered saline, and paraffin embedded. Six μm thick sections were prepared, deparaffinized, and blocked in Coplin jars with 1% bovine serum albumin in 1X phosphate buffer saline for one h at room temperature. The slides were then incubated with a 1:250 dilution of anti-βB2-crystallin antibody (a gift of Dr. Larry Takemoto, Kansas State University) at room temperature for one h and the signal detected using a anti-rabbit Vectastain ABC horseradish peroxidase kit (Vector Laboratories, Burlingame, CA) using diaminobenzidine as a substrate.

## Results

### Quantitative fertility analysis of homozygous *Crybb2^Phil^* mice

Mice homozygous for the *Crybb2^Phil^* mutation were recovered from cryopreserved stocks held by the Animal Genetics Resource, National Institutes of Health, and found to be poor breeders. The fertility/fecundity of these animals was assessed quantitatively in comparison to the outbred Swiss Webster strain that the *Crybb2^Phil^* mutation arose on (Table 1). Overall, STOCK *Crybb2^Phil^* matings were more likely to be sterile and the fertile matings produced smaller litters with fewer pups per litter than the Swiss Webster controls. Crosses between homozygous STOCK *Crybb2^Phil^* and Swiss Webster mice demonstrated that this subfertility was derived from both the male and female (Table 2). Since mice harboring the *Crybb2^Phil^* mutation exhibit autosomal dominant cataract, the fertility of the F1 generation of the Swiss Webster-*Crybb2^Phil^* matings (heterozygous for the *Crybb2^Phil^* mutation) was also assessed and was found to not be significantly different from Swiss Webster controls (data not shown).

**Table 1:** Homozygous STOCK *Crybb2^Phil^* mice are subfertile.

	***Cryhh2*^+^M x *Crybb2*^+^F**	***Crybh2^Phil^*M x *Crybb*f*^Phil^*F**
**Mean Litter Size** (# of pups)	1 1.6±3.2	7.9±4.0
**# of Litters**	41	22
**Mean Fecundity** (pups/30days)	9.2±4.8	4.6±3.6
**# of matings**	19	15
**# of sterile matings**	1 (5%)	3 (20%)
**# of matings producing only one litter**	3(16%)	7 (47%)

*Crybb2^+^* M x *Crybb2^+^* F versus *Crybb2^Phil^* M x *Crybb2^Phil^* F: Litter size p value=0.0002 Fecundity p value=0.0046.

**Table 2:** Both male and female homozygous STOCK *Crybb2^Phil^* mice are subfertile.

	***Crybh2*^+^M x *Crybh2*^+^F**	*Cryhb2^Phil^*M x *Cryhb2+F	*Crybb2*^+^M x Crybb2*^Phil^*F*
**Mean Litter Size** (# of pups)	12.7±2.5	9.5±3.4	7.9±2.0
**Mean Fecundity** (pups/30days)	12.8±4.0	5.4±4.2	4.4±4.0
**N** (# of matings)	8	8	5
**# of sterile matings**	0	2 (25%)	0
**# of matings producing only one litter**	0	2 (25%)	2 (40%)

*Female STOCK *Crybb2^Phil^* homozygotes are not more significantly affected than male STOCK *Crybb2^Phil^* homozygotes (Litter size p value=0.2231; Fecundity p value=0.6685) Litter size: *Crybb2^+^* M x *Crybb2^+^* F versus *Crybb2^Phil^* M x *Crybb2^+^* F: p value=0.003 *Crybb2^+^* M x *Crybb2^+^* F versus *Crybb2^+^* M x *Crybb2^Phil^* F: p value lt 0.001 Fecundity: *Crybb2^+^* M x *Crybb2^+^* F versus *Crybb2^+^* F x *Crybb2^Phil^* M: p value=0.003 *Crybb2^+^* M x *Crybb2^+^* F versus *Crybb2^+^* M x *Crybb2^Phil^* F: p value=0.004.

### Assessment of testis morphology

**Figure 1:**
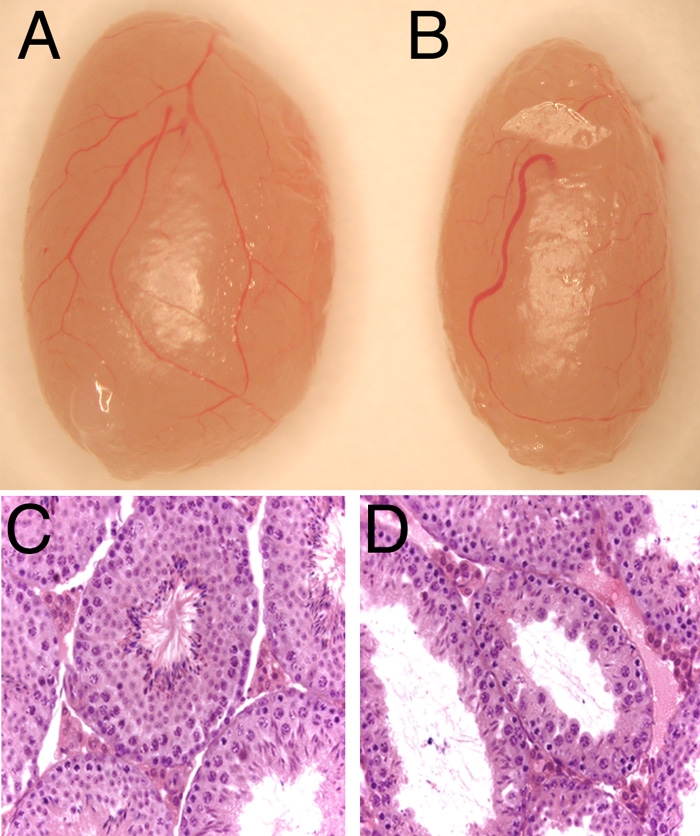
Defects in homozygous STOCK *Crybb2^Phil^* testis. **A**: Gross appearance of a Swiss-Webster mouse testis. **B**: Gross appearance of a STOCK *Crybb2^Phil^* testis. **C**: Hematoxylin and eosin stained paraffin section of a Swiss-Webster mouse testis. **D**: Hematoxylin and eosin stained paraffin section of a homozygous STOCK *Crybb2^Phil^* testis.

Testes from homozygous STOCK *Crybb2^Phil^* mice were observed to be smaller than Swiss Webster controls (Figure 1A,B) and weighed 210±30 mg (N=34) as compared to 250±30 mg (N=28) for the Swiss Webster testis (p less than or equal to 0.0001). This reduction in testis weight was accompanied by the presence of numerous abnormal seminiferous tubules in the STOCK *Crybb2^Phil^* testis (Figure 1C,D). Morphometric analysis of the testis during development revealed that the first abnormality in testis anatomy was a reduction of seminiferous tubule diameter at 1 and 2 weeks of age although STOCK *Crybb2^phy^* seminiferous tubules are larger than normal by 4 weeks of age (Table 3). Sperm counts from the cauda epididymides of 12-week-old mice revealed that homozygous STOCK *Crybb2^phil^* mice had lower sperm counts (2.5±1.8x10^7^ sperm per two cauda (N=26)) than Swiss Webster mice (4.4±1.5x10^7^ sperm per two cauda [N=23]), p less than or equal to 0.0003. Significantly higher numbers of apoptotic developing sperm were detected in the seminiferous tubules of homozygous STOCK *Crybb2^Phil^* mice beginning at 3 weeks of age. The extent of apoptosis diminished by four weeks of age while no statistically significant difference was found at 18 weeks (Table 4). Further, only 59.6±22.8% of sperm from STOCK *Crybb2^phil^* homozygotes (N=15) were motile as compared to 78.3 pom 11.3% of Swiss Webster sperm (N=18).

**Table 3:** Morphometric analysis of seminiferous tubule diameter of Swiss Webster and STOCK *Crybb2^Phil^* homozygous mice.

	*Crybb2^+^*	*Crybb2^Phil^*	p-value
Newborn	52.4±6.5	51.6±7.3	13.83
1 week	66.5±7.1	59.9±6.2	2.3 x 10-9
2 week Mean	85.5±8.7	69.3±7.3	8.07 x 10-31
4 week Mean	161.9±18.5	177.7±23.8	1.36 x 10-5

At birth, the seminiferous tubule diameter (in μm) of wildtype and *crybb2^phil^* mice was not statistically different. However, at one and two weeks postnatal when spermatogenesis is beginning, the tubule diameter of mutants is significantly smaller than wildtype. By four weeks of age, mutant seminiferous tubules become dilated relative to wildtype similar to the situation seen in adult animals.

**Table 4:** Apoptosis detection in seminiferous tubules of Swiss Webster and STOCK *Crybb2^Phil^* homozygous mice.

	% of tubules with at least 1 apoptotic cell		p value
	*Crybb2^+^*	*Crybb2^Phil^*	
2 week	11.5%	13.3%	0.30
3 week	12.3%	23.4%	0.00082
4 week	19.3%	32.2%	0.015
18 week	15.6%	11.9%	0.28

Apoptosis rates (as scored by TUNEL assay) were measured in tissue sections from postnatal testes and were found to be significantly elevated at 3 and 4 weeks of age as the first mature sperm are being produced. By 18 weeks of age, no significant differences were found in the number of TUNEL positive nuclei between wildtype and mutant testes although mutant testes are morphologically abnormal at this age.

### Expression of βB2-crystallin in the testis and sperm

**Figure 2:**
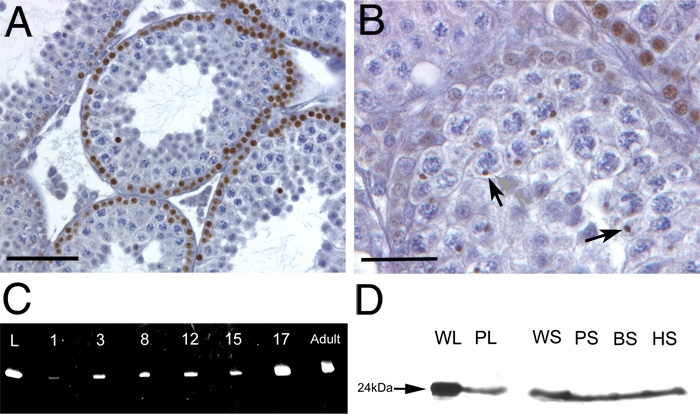
βB2-crystallin protein is expressed in the testis and mature sperm. **A**, **B**: βB2-crystallin localization in the 4 week old mouse testis. **A**: βB2-crystallin (brown stain) is found at the basal surface of some but not all seminiferous tubules in cells having the round nuclei and scant cytoplasm diagnostic of the leptotene and zygotene spermatocytes. **B**: βB2-crystallin is only weakly detected in basal cells with ovoid nuclei characteristic of spermatogonia, but is easily observed in rounded acrosomal vesicles in contact with the nuclei of spermatids (arrowheads). **C**: Semi-quantitative rt-PCR analysis of βB2-crystallin mRNA expression in the testes during postnatal development. L-lens, 1,3,8,12,15,17, age of testis in days post natal. **D**: Western blot analysis of βB2-crystallin protein expression in sperm. WL-wildtype lens, PL- homozygous *Crybb2^Phil^* lens, WS- wildtype mouse sperm, PS- homozygous *Crybb2^Phil^* mouse sperm, BS- bovine sperm, HS- human sperm. **A**, bar=200 μm; **B**, bar=70 μm.

Previous studies have purified βB2-crystallin protein from the rat testis [9], although neither the developmental expression profile nor the cellular distribution of this protein was reported. By immunohistochemistry using a polyclonal antibody raised against βB2-crystallin, we detected βB2-crystallin immunoreactivity in the basal cell layer of some but not all seminiferous tubules in cells that have the round nuclei and scant cytoplasm diagnostic of the leptotene and zygotene spermatocytes [22] (Figure 2A,B) while βB2-crystallin was only weakly detected in basal cells with ovoid nuclei characteristic of spermatogonia (Figure 2B). βB2-crystallin immunoreactivity is also observed in Golgi/rounded acrosomal vesicles in contact with the nuclei of spermatids (Figure 2B), in addition to the head of some but not all maturing sperm (data not shown).

RNA samples were prepared from day 1, 3, 8, 12, 15, 17, and adult Swiss-Webster testis and analyzed by semi-quantitative RT-PCR using βB2-crystallin-specific primers. βB2-crystallin transcripts were detected in the testis from birth throughout postnatal life (Figure 2C). Notably, there is a slight increase in βB2-crystallin expression at postnatal day three, which corresponds to some of the spermatogonia undergoing mitosis [23]. Additionally, there is an upregulation of βB2-crystallin expression at postnatal day 17, which corresponds to the beginning of Meiosis II [23]. Western blot analysis of sperm isolated from the epidydimides of Swiss Webster mice, as well as bovine and human ejaculate confirm the presence of βB2-crystallin protein in sperm in diverse mammals (Figure 2D). Note that βB2-crystallin levels are reduced in the *Crybb2^Phil^* homozygous lens as previously reported [12] while βB2-crystallin levels in sperm from homozygous mutant animals are comparable to those seen in Swiss Webster mice.

### Assessment of ovarian morphology and function

**Figure 3:**
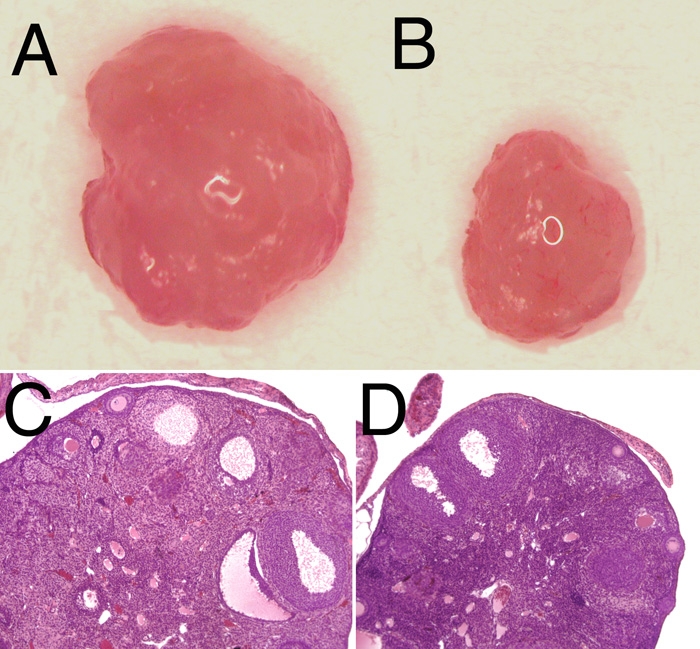
Defects in homozygous STOCK *Crybb2^Phil^* ovary. **A**: Gross appearance of a Swiss-Webster mouse ovary. **B**: Gross appearance of a STOCK *Crybb2^Phil^* ovary. **C**: Hematoxylin and eosin stained paraffin section of a Swiss-Webster mouse ovary. **D**: Hematoxylin and eosin stained paraffin section of a homozygous STOCK *Crybb2^Phil^* ovary.

Ovaries were harvested from Swiss-Webster and STOCK *Crybb2^Phil^* homozygous mice and viewed under a dissecting microscope revealing an evident difference in ovary size (Figure 3A,B). Further, quantitative determination of ovary weights demonstrated that the STOCK *Crybb2^Phil^* homozygotes have a 70% reduction in ovarian mass. Mean ovarian weight for wild type mice (N=4) was 9.7 mg±2.1 mg while *Crybb2^Phil^* homozygotes (N=4) averaged 2.9±1.9 mg, p less than or equal to 0.0001. Histological analysis indicated that this reduction in ovarian size was due to a reduction in the number of developing follicles (Figure 3C,D). This was tested by assessing the ability of 12 week old Swiss Webster and STOCK *Crybb2^Phil^* homozygotes to respond to superovulation with exogenous gonadotropins. Swiss-Webster mice produced an average of 25.5 eggs (N=4) while STOCK *Crybb2^Phil^* homozygous mice produced an average of 1.6 eggs (N=5), p less than or equal to 0.0006. Both rt-PCR and western blotting detected βB2-crystallin expression in the adult ovary (data not shown), although attempts to localize this protein in the ovary were inconclusive.

### Assessment of fertility in male C57Bl/6NHsd-*Crybb2^Phil^* mice

While the current data indicate that βB2-crystallin plays a role in male and female fertility, there is still the possibility that the subfertility observed in STOCK *Crybb2^phil^* mice arose from inbreeding depression occurring over the 20-30 years these animals were isolated from the original outbred Swiss Webster line or from a new mutation in this strain. Thus, we moved the *Crybb2^Phil^* mutation onto the inbred C57Bl/6NHsd strain by 8-10 generations of backcrossing, then reestablished homozygosity by intercrosses. Male mice on the C57Bl/6NHsd background either wildtype or homozygous for the *Crybb2^Phil^* mutation were then mated to ICR females and their reproductive performance assessed over a three month period (Table 5). In contrast to the STOCK *Crybb2^Phil^* mice, no statistically significant impairment of fecundity was noted in *Crybb2^Phil^* animals on the C57Bl/6NHsd background as compared to control C57Bl/6NHsd animals.

**Table 5:** Fertility is not impaired in homozygous male C57Bl/6NHsd-*Crybb2^Phil^* mice.

	***Crybb2*^+^M x *Crybb2*^+^F**	***Crybb2^Phil^*M x *Crybb2*^+^F**
**Mean Litter Size** (# of pups)	13.4±4.2	12.8±1.9
**Mean Fecundity** (pups/30days)	8.9±5.8	5.9±4.6
**N** (# of matings)	5	5
**# of sterile** **matings**	1 (20%)	0
**# of matings** **producing only one litter**	0	4 (80%)

The fertility of male mice harboring the *Crybb2^Phil^* mutation was assayed by mating adult animals with ICR females for 90 days and the litter size and overall fecundity (pups produced per 30 day period) was assessed. No significant differences were found in either litter size or fecundity for the small N (5 matings) although mutant males produced fewer litters of pups overall than wildtype males.

## Discussion

Previous studies have demonstrated that βB2-crystallin mRNA and protein are present in the testis [9], although its cellular distribution and function were not described. Here we demonstrate that βB2-crystallin is a component of mammalian sperm and is present in developing sperm from the spermatocyte stage onward. In order to identify the function of βB2-crystallin in the testis, we then assessed the fecundity of mice harboring an in-frame 12 nucleotide deletion which would be expected to disrupt the structure of the fourth "Greek key" motif of the protein [19] which is necessary for its normal association properties [24]. In lenses, this mutation has been demonstrated to result in insolubility of βB2-crystallin [25] and the development of autosomal dominant cataract [20].

Fertility analysis of *Crybb2^Phil^* mice on the original Swiss Webster derived genetic background demonstrated the both male and female homozygous *Crybb2^Phil^* mice are subfertile, suggesting that βB2-crystallin plays an important role in testis and ovary function. In the testis, the fertility defect was traced to reductions in both epidydimal sperm counts resulting from increased apoptosis of developing sperm and sperm motility. Interestingly, it was recently reported that βB2-crystallin copurifies with microtubules isolated from lens epithelial cells, leading to the hypothesis that βB2-crystallin is a microtubule associated protein that may protect microtubules from denaturation [26]. Since microtubules are essential for sperm motility [27], this may suggest a functional connection between mutation of βB2-crystallin and infertility. In the ovary, the number of developing follicles was reduced in homozygous *Crybb2^Phil^* mice, resulting in drastic reductions in the number of eggs produced in response to superovulation. Overall, these data suggest an important role for βB2-crystallin in the maturation of both eggs and sperm.

In contrast, no defects were observed in either the testis or ovary of homozygous *Crybb2^Phil^* mice on the C57Bl/6NHsd genetic background (data not shown). Further, no reductions in fecundity were noted in homozygous male mice on this strain. There are at least three possible explanations for these conflicting results. First, the *Crybb2^Phil^* mutation arose spontaneously in a breeding colony of outbred Swiss-Webster mice [20] and was then housed for over 20 years in a small breeding colony with no attempt to maintain the original genetic diversity of the strain. Although no directed attempts at inbreeding were made, it is very likely that the genetic diversity found in the founder colony was largely lost during this husbandry period. Since inbreeding has been associated with impaired reproductive fitness due to reductions in testicular mass, sperm concentration, and sperm quality in both wild rabbits and old field mice [28,29], inbreeding of the founding *Crybb2^Phil^* population is a plausible explanation for the reduced fecundity of STOCK *Crybb2^Phil^* mice in comparison to outbred Swiss Webster controls. Second, cryopreservation of STOCK *Crybb2^Phil^* embryos may have caused a de novo mutation in a recessive gene required for fertility. In these cases, the subfertility noted in STOCK *Crybb2^Phil^* mice is unrelated to the presence of the *Crybb2^Phil^* mutation and instead results from either a new independent mutation affecting fecundity or the loss of heterozygosity at numerous loci as has been observed in endangered wildlife [30]. Conversely, it is possible that βB2-crystallin is important for sperm and egg production but the C57Bl/Hsd strain harbors a genetic locus able to suppress the effect of this mutation.

Overall, this study has demonstrated that βB2-crystallin protein is present in mature mammalian sperm and its expression pattern in the testis is consistent with a role in sperm development. We have also demonstrated that both male and female homozygous STOCK *Crybb2^Phil^* mice are subfertile due to defects in sperm and egg production, although *C57Bl/6NHsd-Crybb2^Phil^* homozygotes have apparently normal gonads. It is interesting to note that all known mutations in the βB2-crystallin gene in both mice and humans disrupt only the fourth Greek key of the protein and result in dominant cataracts [19,31-35]. Since no complete null mutation in the βB2-crystallin gene has ever been identified, definitive elucidation of the extralenticular function of βB2-crystallin will await characterization of targeted knockout mice.
